# Perceptions of Pharmacy Graduate Students Toward Research Ethics Education: A Cross-Sectional Study from a Developing Country

**DOI:** 10.1007/s11948-022-00406-0

**Published:** 2022-10-26

**Authors:** Wesam S. Ahmed, Amgad Ahmed, Karem H. Alzoubi, Camille Nebeker

**Affiliations:** 1 College of Health and Life Sciences, Hamad Bin Khalifa University, Qatar Foundation, Doha, Qatar; 2 Division of Orthodontics, Department of Preventive Dentistry, Faculty of Dentistry, Jordan University of Science and Technology, Irbid, Jordan; 3 Department of Pharmacy Practice and Pharmacotherapeutics, College of Pharmacy, University of Sharjah, Sharjah, 27272 United Arab Emirates; 4 Department of Clinical Pharmacy, Faculty of Pharmacy, Jordan University of Science and Technology, Irbid, 22110 Jordan; 5 Herbert Wertheim School of Public Health and Human Longevity Science, University of California San Diego, La Jolla, CA USA

**Keywords:** Jordan, Pharmacy, Education, Middle East and North Africa (MENA), Research ethics, Responsible conduct of research (RCR)

## Abstract

Despite the potential value of graduate-level research ethics training, most Middle East countries, including Jordan, do not routinely offer formal research ethics training. In students enrolled in Jordanian master’s level graduate program in pharmacy, the current study assessed: 1- differences in pre- and post-enrollment exposure to research ethics core themes, 2- whether this exposure was through a formal course or in an informal setting, and 3- student attitudes towards research ethics education and the need for integrating a dedicated research ethics course into pharmacy graduate programs. A 12-item on-line survey was developed by the authors and disseminated to a convenience sample of current and former master-level pharmacy students in Jordan. A total of 61 eligible respondents completed the survey. A minority of respondents (38%) acknowledged receiving research ethics training prior to enrollment into a postgraduate pharmacy program with nearly half (16%) describing this training as informal. In comparison, a larger percentage of the total respondents (56%) had received research ethics training during their postgraduate program enrollment, with nearly half of those (25%) indicating that this training was informal. A majority of respondents reported a strong need for integrating a formal research ethics course into postgraduate pharmacy curriculum (90%) to support their research training and thesis writing (89%). Overall, the study revealed a notable lack of research ethics education for graduate-level pharmacy students in Jordan.

## Introduction

Most countries of the Arab Middle East and North Africa (MENA) region lack national mandates requiring education in the responsible conduct of research (RCR). Increased cases of research misconduct in the 1970’s led the United States (US) National Institute of Health (NIH) to introduce a federal requirement for RCR education for trainees engaged in biomedical research. The regulation stated expectations for research organizations to foster research integrity by creating opportunities for the research community to be aware of accepted norms and social responsibilities (Albertsa et al., 2014; Antes et al., 2009; Kalichman, 2013, 2016; Kalichman & Plemmons, 2018; Steneck & Bulger, 2007). The NIH was among the first research sponsors to call for RCR training in 1989 (NIH, 1989). These requirements were further refined through updated versions of the NIH training mandate that followed in 1992, 2000, and finally 2009 (NIH, 1992, 2000, 2009). The NIH defined RCR training expectations in its 2009 update as the application of ethical principles in the practice of all research-related activities dictated by relevant professional norms (NIH, 2009). Furthermore, the NIH requirements specified topics to be discussed including ethics of research involving humans and animals, conflict of interest, data management, research misconduct, and others (Kalichman, 2013). Additionally, The US federal Office of Research Integrity (ORI) published nine core areas^1^ to be addressed in an RCR course. These core topic areas were further expanded in a Delphi consensus panel report that identified 53 topics in seven core areas that could be included in RCR teaching (DuBois & Dueker, 2009; NIH, 2009).

While the US was advancing RCR training through federal training mandates, researchers were beginning to explore RCR instructional goals and evaluating the potential value of RCR training (AlMahmoud et al., 2017; Antes et al., 2009; Mulhearn et al., 2017; Plemmons et al., 2006; Watts et al., 2017). While clearly a priority in the US, RCR training expectations remain nonextant in most Middle East countries including Jordan (Alahmad et al., 2012). Jordan is a country of the MENA region with a progressive clinical research agenda (Ahmed et al., 2020) and the first Arab country to enact clinical research regulations (Al-Omari & Al-Hussaini, 2017). The country has been chosen as a hub for the Research Ethics Program in Jordan,^2^ an initiative that is supported by a research grant from the US NIH and targets early career researchers and senior graduate students from the Arab MENA region with the aim of promoting RE in the region (Al-Shami et al., 2022). Although Institutional Review Board (IRB) approval is required for all research involving human subjects, the country has no RCR educational expectations for students and researchers, regardless of the funding source. Training in RCR is an important aspect of postgraduate education, especially for those who are doing research as part of their postgraduate degree requirement (Peiffer et al., 2008). Similar to most undergraduate health science programs in the Middle East, undergraduate pharmacy programs do not require completion of a research project (Al-Wazaify et al., 2006). As such, it is safe to assume that newly enrolled graduate students will have little, if any, prior exposure to learning about RCR topics. It follows that there is a need for RCR training early in graduate education as part of the core curriculum. As many graduate students concurrently work as research assistants in research laboratories, RCR education becomes an important foundation for fostering awareness of accepted norms and conventions that support research integrity. Moreover, Jordan has a well-recognized pharmaceutical industry that leans on contract research organizations for drug development and post-marketing studies. In fact, this pharmaceutical sector exports about 80 percent of its production to more than 60 countries globally, making it the second largest exporting industry in the country (Sweis et al., 2015). Indeed, the research and development in this pharmaceutical industry relies on pharmacy graduates, which provides another reason why RCR education is important for this specific group.

In a recent content analysis study of pharmacy program course offerings in Jordan (Ahmed & Nebeker, 2021), 10 thesis-track master pharmacy programs were identified. Thesis-track master programs are programs that have both coursework and research completion requirements. These programs were distributed across seven universities in Jordan (Ahmed & Nebeker, 2021). Of the 10 programs, none reported having a standalone RCR course integrated within the curriculum and only four reported discussions of RCR topics integrated within one of the program’s courses. The RCR topics of focus were primarily human and animal research protections. Three programs were offered by Jordan University of Science and Technology (JUST), and one program by the University of Jordan (JU) (Ahmed & Nebeker, 2021). These two universities are the highest ranked universities in Jordan with high research output.^3^ The University of Jordan is also the oldest university in Jordan (Tarboush et al., 2020). Our study was designed to assess exposure to RCR content by current students and recent graduates of pharmacy master programs at the aforementioned two universities. Specifically, we were interested in topics covered, whether exposure occurred through formal or informal settings, and whether there was a difference in exposure prior and post enrollment into a postgraduate program. In addition, we assessed participant attitudes towards RCR education and whether they believe there is a need for integrating a standalone RCR module into program curricula.

## Methods

### Questionnaire Design

The current study of pharmacy graduate students assessed: 1- differences in pre- and post-enrollment exposure to RCR core themes, 2- whether this exposure was through a formal course or in an informal setting, and, 3- student attitudes towards RCR education and the need for integrating a dedicated RCR course into pharmacy graduate programs. To achieve this, a 12-item survey (Appendix A) was constructed and deployed online over a two-week period (Dec 1st to Dec 15, 2017) through “suveymonkey.com” website. The survey was administered in English, since English is the language used in graduate programs offered at JUST and JU. The survey utilized several response formats including multiple choice, “Yes”, “No”, or “I do not know”, multiple checkbox, and open-ended items. The survey consisted of three sections with the first section soliciting demographic information from participants, including the name of the postgraduate pharmacy program, the university at which it is offered, and the location of the university. Demographic questions also focused on whether the participant had recently completed the program or was currently enrolled and, if the latter was true, the academic year of current enrollment. Lastly, university and country where participants had received their undergraduate pharmacy education were requested.

The second section assessed participants’ prior and current exposure to RCR core areas as described by the US ORI^4^ and NIH, 2009 updated mandates (NIH, 2009), whether this exposure was formal or informal, and the topics covered. RCR training was defined as training that addressed the ethical dimensions of planning, conducting, and reporting of research. Formal training was outlined as any course (online, face-to-face, or combination of the two) that the participant had enrolled in and completed. Informal training consisted of opportunities to discuss ethical dimensions of research with a supervisor, mentor or lab meeting or mentioned in a course but, not the primary focus of the course.

The last section of the questionnaire was designed to solicit the participants’ opinion towards RCR education as a tool in preparing trainees to conduct research and to write their thesis, as well as their thoughts towards implementing a standalone RCR course into pharmacy postgraduate programs.

The questionnaire was revised by a group of experts. Their feedback was then implemented in the revised questionnaire. The survey was piloted to a group of eligible participants (n = 7) (Connelly, 2008). Responses from the pilot study were not included in the final data analysis. The study was verified as exempt by the JUST IRB committee (ref. # 13/109/2017).

### Study Participants

The survey was directed to current and former pharmacy graduate students at JU and JUST. The survey description and weblink was disseminated to potential participants through five social media groups (WhatsApp and Facebook) that included graduate pharmacy students or recent alumni of JUST or JU. A recruitment reminder was sent one week after deploying the survey and two days prior to closing the survey. The consent script was included in the introduction at the beginning of the questionnaire (Appendix A) as well as in the recruitment invitation sent through the social media platforms.

### Data Analysis and Figure Preparation

Questionnaires that were not fully completed or that included participants who did not meet the eligibility criteria (those who were not pharmacy master students/graduates, not studying in JU or JUST, or did not have a research completion degree requirement) were excluded from the data analysis. Data were analyzed using Microsoft Excel 13 and SPSS V21. Descriptive statistics were used to summarize the data. Answers to questions were reported as percentages out of the total. Chi square test was used for cross-tabulation of country of undergraduate education, postgraduate university, enrollment status, academic year, and master program with RE exposure prior or during enrollment, and with participants’ opinions about research ethics education. A P-value of less than 0.05 was considered statistically significant.

A priori power analysis was conducted using G*power v3.1.9.7 (Faul et al., 2009) to determine the minimum sample size required to test the study hypothesis. Results indicate the required sample size to achieve 80% power for detecting a large effect (w = 0.5) at a significant criterion of α = 0.05, assuming the highest degree of freedom used in the test (df = 8), was N = 61 for Chi-Squair test. The calculated critical Chi-Squair value and noncentrality parameter λ were 15.507 and 15.250, respectively. The actual (calculated) power was 80.718%. Figures were prepared using Microsoft Excel 13.

## Results

### Respondents’ Demographics

The survey link was deployed to five social media groups. The social media groups collectively included 181 group members, 146 members saw the survey invitation post, which included the link to the survey, and 70 filled out the survey (response rate 48%). Incomplete responses and ineligible participants were excluded from data analysis, leaving a total of 61 eligible participants who completed the survey (completion rate of 87%). All participants had received (41%, n = 25) or were completing (59%, n = 36) their pharmacy master education at either JU or JUST. All had been enrolled in a thesis-track master program with a research completion requirement. The majority (89%, n = 54) received their undergraduate pharmacy education in Jordan, mostly in JU (30%, n = 18) and JUST (48%, n = 29). Most participants (59%, n = 36) were currently enrolled in a pharmacy master program, of whom the majority were in their third year (28%, n = 17). Most participants were enrolled in Clinical Pharmacy (38%, n = 23) and Pharmaceutical Sciences (36%, n = 22) master programs (Table 1).

**Table 1:** Demographic characteristics of participants

Variable	N (%)
*Undergraduate education*	
Jordan	54 (88.5)
The University of Jordan (JU)	18 (29.5)
Jordan University of Science and Technology (JUST)	29 (47.5)
Amman Al-Ahliyya University	4 (6.6)
Applied Science University	1 (1.6)
Philadelphia University of Jordan	1 (1.6)
University of Petra	1 (1.6)
Other countries	7 (11.5)
Al-Azhar University (Palestine)	1 (1.6)
Damascus University, (Syria)	1 (1.6)
Kuwait University (Kuwait)	1 (1.6)
Sana’a University (Yemen)	1 (1.6)
Dhamar University (Yemen)	1 (1.6)
Aden University (Yemen)	2 (3.3)
*Postgraduate education*	
Jordan	61 (100)
JU	38 (62.3)
JUST	23 (37.7)
Other countries	0 (0)
*Enrollment*	
Graduated	25 (41)
Currently enrolled	36 (59)
First year	8 (13.1)
Second year	9 (14.8)
Third year	17 (27.9)
Fourth year	2 (3.3)
Enrolled in a thesis-track master program	61 (100)
* Master program*	
Pharmaceutical Sciences	22 (36.1)
Clinical Pharmacy	23 (37.7)
Pharmaceutical Technology	5 (8.2)
Medicinal Chemistry	4 (6.6)
No answer	7 (11.4)

### Exposure to RCR Education Prior to Master’s Degree Enrollment

**Fig. 1:**
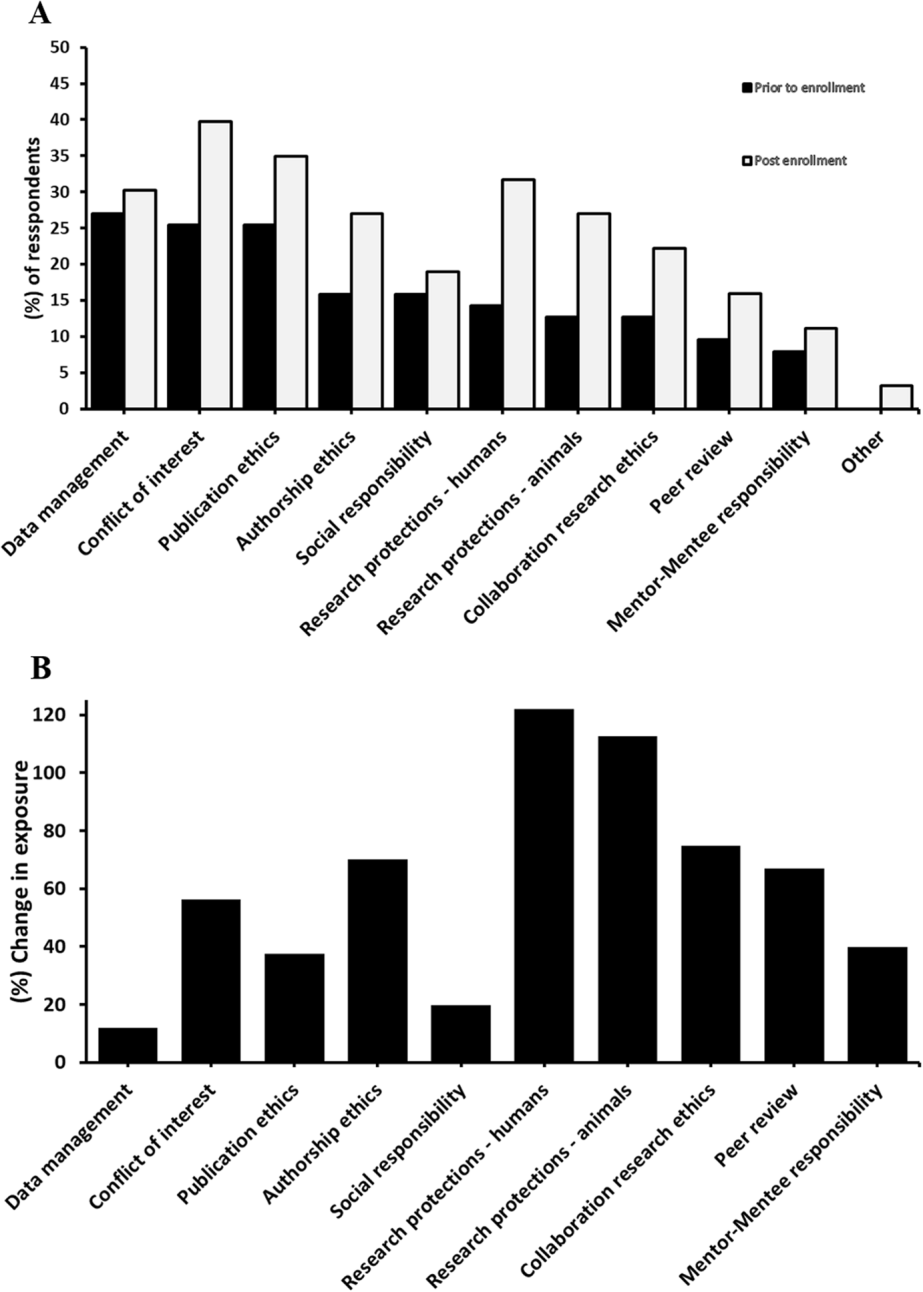
Research ethics core areas covered prior and during enrollment into a master’s degree program (**A**) and the percent change in participants’ exposure to these core areas after enrollment (**B**). N = 61

The majority of the participants (62%, n = 38) had not received RCR education prior to enrollment into a master’s degree program. Those who had received prior RCR training (38%, n = 23) reported mostly informal training (16%, n = 10). Cross tabulation of prior RCR training with undergraduate university, post-graduate university, enrollment status, current academic year, and master program, each separately, did not reveal a statistically significant difference between the groups (*P* value > 0.05) (Table 2). Prior training content focused on data management (28%, n = 17), conflict of interest (26%, n = 16), and publication ethics (26%, n = 16). Respondents reported little prior training on mentor–mentee responsibility (8%, n = 5) or peer review ethics (10%, n = 6) (Fig. 1A).

**Table 2:** Cross-tabulation of the country of undergraduate education, postgraduate university, enrollment status, academic year, and master program with answers to questions 7, 9, 11, and 12

Item	N (%)	*P* value				
		Undergrad university (In Jordan vs outside of Jordan)	Postgrad university	Enrollment	Academic year	Master program
*RE training prior to enrollment*						
Received	23 (37.7	1.000	0.652	0.055	0.351	0.763
Formal (online)	2 (3.3)					
Formal (face-to-face)	8 (13.1)					
Informal	10 (16.4)					
Combination (formal and informal)	3 (4.9)					
Not received	38 (62.3)					
*RE training during enrollment*						
Received	34 (55.7)	0.628	0.013*	0.263	0.253	0.029*
Formal (online)	4 (6.6)					
Formal (face- to-face)	13 (21.3)					
Informal	15 (24.6)					
Combination (formal and informal)	2 (3.3)					
Not received	27 (44.3)					
(Q11) Could a RE course help in performing research and thesis writing?		0.594	0.489	1.000	0.306	0.146
Yes	54 (88.5)					
No	4 (6.6)					
I do not know	3 (4.9)					
(Q12) Is there a need to integrate a dedicated research ethics course into postgraduate pharmacy programs?		0.535	0.521	0.524	0.268	0.159
Yes	55 (90.2)					
No	4 (6.6)					
I do not know	2 (3.3)					

*Refers to *P* value less than 0.05

### Exposure to RCR Education During Master’s Degree Enrollment

Results indicated that 56% (n = 34) of respondents received some form of RCR education during their enrollment, where 25% (n = 15) of this was informal training and 18% (n = 17) was formal education (Table 2). Cross tabulation of post-enrollment RCR education/training exposure with undergraduate university, post-graduate university, enrollment status, current academic year, and master program, each separately, revealed a statistically significant difference between post enrollment RCR exposure and post-graduate university (*P* = 0.013) and master program (*P* = 0.029). Conflict of interest (41%, n = 25), publication ethics (36%, n = 22), and human subjects protections (33%, n = 20) were the topics most frequently discussed, whereas mentor–mentee responsibility (12%, n = 7) and peer review (16%, n = 10) were the least discussed topics (Fig. 1B).

### Respondents’ Opinions

The majority of the respondents affirmed that receiving a formal RCR education would help in performing better in research projects and in thesis writing (89%, n = 54). Most respondents (90%, n = 55) believe that integrating a dedicated RCR module into the curriculum of the master programs is important (Table 2).

Participants who supported integrating a standalone RCR course justified their answer as follows:

- “It helps to be more oriented”
- “It helps in doing research more ethically”
- “Because no course is found in the program to cover this part”
- “It is a must”
- “It is important to take into consideration the ethical issues while doing research”
- “Ethics are important for doing good confidential research that helps society to grow”
- “It facilitates the conduct of research and the completion of our research project”
- “It is needed in our field of research”
- “It would be very helpful”

Those who did not support the idea defended their point of view as follows:

- “One comprehensive lecture or workshop is enough”
- “I was fortunate enough to have been taught about RE by my supervisor, however, not everyone gets this opportunity, mainly due to the assumption that this was taught during the BSc degree, or because the subject “RE” is basic in one’s understanding. Having said that, I do not believe a course is needed, but a 1–2 hour workshop should be enough for postgrad students where the highlights are given to the students as well as a reference to read more about it”
- “I think the research methodology course in our department is enough to cover the ethical issues of research”

## Discussion

The current study reveals a lack of exposure to RCR core themes prior and post-enrollment into graduate pharmacy programs, with pre-enrollment exposure being even less than post-enrollment. However, there was a relative increase in the exposure to all RCR core areas post-enrollment, with the highest increase in exposure noticed for topics that were formally discussed in mandatory courses (Ahmed & Nebeker, 2021), indicating the effectiveness of integrating formal RCR discussions in enhancing graduate student’s exposure to research ethics topics. Moreover, there was a consensus among the participants on the value formal RCR education offers (88.5%, n = 54), who also expressed the need for integrating a dedicated course into the programs curriculum that covers most core RCR areas relevant to their field of study (90.2%, n = 55).

Questionable research practices (QRPs) may occur during graduate education and can be difficult to resolve due to lack of clarity in definitions and limitations in student’s decision making. As such, to minimize the risk of both misconduct and QRPs, principal investigators need to address relevant scientific norms as well as what constitutes research misconduct with students (Resnik and Stewart Jr, 2012). In addition, underscoring the role of culture in adhering to the best ethical practices highlights the importance of early training in the RCR principles for both students and researchers (Davis, 2003). For that matter, it seems unethical conduct in research is a shared commodity in both developed and developing countries. In one study, research misconduct in low and middle income countries was reported to be as common there as in high income countries (Ana et al., 2013). Therefore, education in the RCR would seem a global language in responding to QRPs, research misconduct and in fostering research integrity and promoting positive attitudes in research.

The present study gives insight into the exposure and attitudes of pharmacy master students and graduates towards RCR education in the two major and top ranked universities in Jordan. The study revealed that only a minority (38%, n = 23) of the participants received some sort of RE training prior to their enrollment into a master’s degree program. This was regardless of whether they received their undergrad education inside or outside of Jordan. Almost half of this training (16%, n = 10) was informal. Undergraduate pharmacy programs in Jordan and most of the Middle East countries do not have a research project completion requirement (Al-Wazaify et al., 2006). Therefore, it is expected that most undergraduate students do not get exposed to formal RCR training during their undergraduate degree enrolment. Nonetheless, there are few optional research-oriented venues, such as students research clubs, where students can opt in to join. Therefore, it might be that those who reported prior-to-enrollment knowledge in some RCR topics have obtained such training through these venues. Importantly, data management (28%, n = 17), conflict of interest (26%, n = 16), and publication ethics (26%, n = 16) were the topics participants reported being most familiar.

Comparatively, more participants (56%, n = 34) reported receiving research ethics education during their enrollment into a graduate program, indicating an overall increase in the participants’ exposure to research ethics concepts post-enrollment. Analyzing the participants’ exposure to different RCR core areas revealed a post-enrollment increase in the exposure to a variable degree. The percent increase in exposure was the highest for such topics as human research protections (121% increase), animal research protections (113% increase) (Fig. 1B). These topics were previously identified as the main focus of research ethics discussions in these master programs (Ahmed & Nebeker, 2021). Additionally, more modest, but relatively noticeable, exposure to other topics such as collaboration research ethics (75% increase), authorship ethics (70% increase), peer review (67% increase), and conflict of interest (56% increase) was observed. As many of these topics are not the main focus of the research ethics discussions in these master programs (Ahmed & Nebeker, 2021), it is possible that exposure to these core topics occurred through informal settings. In fact, a large percentage (25% of the total responses, n = 15) of post-enrollment RCR exposure was described as being purely informal. Participants may have been exposed to this informal training after engaging with their thesis projects, as 41% (n = 25) and 28% (n = 17) of the participants were graduates and third-year students, respectively. On the other hand, the least change in exposure was observed for such topics as mentor–mentee responsibility (40% increase), publication ethics (37% increase), social responsibility (20% increase), and data management (12% increase), indicating no large difference in exposure to these topics from pre-enrollment into the master programs (Fig. 1B). More importantly, a cross-tabulation of post-enrollment overall exposure to RCR core areas with the graduate university where students were enrolled revealed a statistically significance difference between the two universities (*P* = 0.013). This disparity can be explained as all master pharmacy programs offered by JUST (Clinical Pharmacy, Pharmaceutical Technology, and Medicinal Chemistry) were reported to integrate some discussions of RE themes into their “Research Methodology” mandatory course (Ahmed & Nebeker, 2021). However, the school of pharmacy at JU offers a “Clinical Pharmacy” master program that incorporates RE discussions into its “Clinical Research Methods and Statistics” course. Moreover, the same school also offers a “Pharmaceutical Sciences” master program that was reported to lack formal discussions of RE themes (Ahmed & Nebeker, 2021). For similar reasons, the cross tabulation with the type of master program revealed a statistically significant difference (*P* = 0.029).

The majority of participants (89%, n = 54) think that a RCR course is important for doing research and aids in thesis writing. About 90% think that there is a need to integrate a standalone RCR course into pharmacy master programs. This perspective was regardless of the university, postgraduate program, enrollment status, or the master program in which the participants were enrolled (*P* > 0.5). In fact, in a recent survey by Tarboush et al. (2020), the majority (87%) of health sciences faculty members in Jordan also supported integrating mandatory research ethics postgraduate course (Tarboush et al., 2020). This attitude of health science faculty members towards integrating RCR mandatory course also suggests that the lack of postgraduate training in research ethics in Jordan extends to graduate programs from health science disciplines other than pharmacy. In further support of this, Swedan et al. (2020) have reported in their multidisciplinary and multi-university survey that included graduate students performing human subjects research in Jordan that only 37% were exposed to RCR education (Swedan et al., 2020). Indeed, this type of RCR training was also deemed necessary for other research investigators, as previous studies in Jordan revealed that the majority (> 80%) of resident doctors, health sciences faculty members, and healthcare investigators have agreed that such training in research ethics is essential for clinical investigators, faculty affiliates, and IRB members in Jordan (Al Demour et al., 2019; Ayoub et al., 2019; Rababa'h et al., 2020; Tarboush et al., 2020), although graduate students reported more unfamiliarity and misconceptions regarding RCR topics (Rababa'h et al., 2020). Even though Jordan’s “Accreditation and Quality Assurance Commission for Higher Education Institutions” oversees that “minimal requirements” are being fulfilled in order to establish any new graduate program, research ethics education does not seem to be part of these “minimal requirements” (Ahmed & Nebeker, 2021). Therefore, integrating research ethics education into graduate pharmacy programs in Jordan is encouraged. Establishing a federal body in Jordan and other middle and low income countries that oversees research integrity—for instance, one with similar missions to the US federal Office of Research Integrity^5^ and the Department of Supervision and Scientific Integrity of China’s Ministry of Science and Technology^6^ may be a tremendous asset for implementing different aspects of research integrity into research institutions and research-based graduate programs and in handling research misconduct allegations (Frankel et al., 2016; Zeng & Resnik, 2010).

Overall, this study revealed a lack of appropriate training (both formal and informal) in core areas of RCR for graduate-level pharmacy students, who would like such training to be a required part of the curriculum. Although discussions in several RCR core areas were missing, our study highlights that integrating ‘formal’ research ethics discussions in a required course could be more efficient in providing more students’ exposure to these core topics compared to informal training. Therefore, given the potential role of RCR training in preserving research integrity (AlMahmoud et al., 2017; Antes et al., 2009, 2010; Plemmons et al., 2006), integrating research ethics education material into pharmacy graduate programs is strongly recommended for programs that lack this training focus. Programs that have already integrated some formal RCR training are encouraged to expand the scope of related discussions to include more core areas and to consider RCR education as a standalone course. As research has shown, RCR programs conducted separately from the standard curricula were likely more successful than those imbedded into existing modules (Antes et al., 2009).

The approach we followed in this study can be extrapolated to graduate programs in Jordan from disciplines other than pharmacy. It can also be utilized and built on by academic institutions from other countries in the region to investigate what research ethics topics -regardless of modality- are covered in graduate programs, how this coverage compares to pre-enrollment exposure to these topics, which modality (formal vs informal) is more effective in promoting student’s exposure to the core themes, and in the assessment of students’ attitude and need for formally introducing a course that covers research ethics main themes.

### Limitations and Future Directions

The current study provides insights based on students’ self-reported recall and may not accurately represent what RCR topics were actually covered, or the extent to which they were covered. A future study involving RCR training instructors may be useful in documenting what topics they cover, the depth of the material covered, and evidence that documents teaching and learning outcomes.

Generally, before enrolling into a research-based degree, RCR training should not be expected, nor it is necessarily needed for pharmacy undergraduate students in Jordan. This lack of expectation of training prior to graduate degree enrollment stems from lack of “research completion” requirement for these undergraduate programs. Nonetheless, our results show that few students have received some sort of research ethics training—mostly at informal settings—prior to graduate degree enrollment. Although we believe that the pre-enrollment, undergrad training might have occurred outside the standard curricula—for instance, through research-oriented student clubs -, there is still room for future investigations. For instance, what are the circumstances where this type of training occurred pre-enrollment, whether there was some aspect of undergraduate program(s) that specially emphasized research readiness, and what might be some reasons for differences in what is covered pre-enrolment vs post-enrollment.

## Conclusion

Education in research ethics core topics is lacking for pharmacy graduate programs in Jordan. In this regard, integrating a formal and dedicated research ethics course into graduate programs in Jordan would seem an effective approach to enhance students’ exposure to these core topics. Informal training is often optional and may impose unequal exposure upon students. In fact, with respect to pharmacy master programs in Jordan, such informal training in the RCR is not a requirement under any circumstances and, in most instances, is not readily available outside the individual-to-individual context. Therefore, although this study revealed that a large percentage of student’s exposure was informal, such informal training may not be an adequate replacement to formal education. Evidence from other studies in Jordan suggest that such deficiency in research ethics training extends to medical residents, students from other disciplines, and even faculty and IRB members. Nonetheless, a comprehensive assessment that includes graduate students from other universities in Jordan and from other health science disciplines may be required to explore how RCR education deficiencies noted in the current study translate to other universities and graduate programs.

## Data Availability

All relevant data are within the manuscript and its appendix.

## Appendix A: Survey Questionnaire

Dear participant,

We invite you to participate in this quick survey. The survey will help us assess the research ethics education offerings in postgraduate pharmacy programs in Jordan. The target group of this survey is postgraduate pharmacy students who are currently receiving or have completed their postgraduate pharmacy education in Jordan. The information provided by you will be used for research purposes. The survey will take about 5 min to complete. All your answers are anonymous and confidential.

Thank you in advance for your participation.

Sincerely,

## Footnotes

1 https://ori.hhs.gov/rcr-objectives-introduction.

2 https://jordanrcrprogram.com.

3 https://www.topuniversities.com/university-rankings/world-university-rankings/2021.

4 https://ori.hhs.gov/rcr-objectives-introduction.

5 https://ori.hhs.gov/.

6 http://en.most.gov.cn/organization/Departments/201809/t20180927_143072.htm.
